# Effects of co-treatment with sulforaphane and autophagy modulators on uridine 5′-diphospho-glucuronosyltransferase 1A isoforms and cytochrome P450 3A4 expression in Caco-2 human colon cancer cells

**DOI:** 10.3892/ol.2014.2536

**Published:** 2014-09-15

**Authors:** MIN WANG, JING-YU ZHU, SHUO CHEN, YING QING, DONG WU, YING-MIN LIN, JI-ZHUANG LUO, WEI HAN, YAN-QING LI

**Affiliations:** 1 Department of Geriatrics and Gastroenterology, Qi-Lu Hospital of Shandong University, Key Laboratory of Proteomics of Shandong, Jinan, Shandong 250012, P.R. China; 2 Department of Gastroenterology, Jinan Central Hospital, Shandong University, Jinan, Shandong 250013, P.R. China; 3 Department of Gastroenterology, China-Japan Friendship Hospital, Beijing 100029, P.R. China; 4 Graduate School, Peking Union Medical College and Chinese Academy of Medical Sciences, Beijing 100029, P.R. China; 5 Department of General Surgery, Qi-Lu Hospital of Shandong University, Jinan, Shandong 250012, P.R. China; 6 School of Medicine, Shanghai Jiao Tong University, Shanghai 200030, P.R. China; 7 Department of Gastroenterology, Qi-Lu Hospital of Shandong University, Jinan, Shandong 250012, P.R. China

**Keywords:** uridine 5′-diphospho-glucuronosyltransferase 1A, Caco-2 cells, sulforaphane, chemoprevention, cytochrome P450 3A4, autophagy

## Abstract

Sulforaphane (SFN), which is highly enriched in cruciferous vegetables, has been investigated for its cancer chemopreventive properties and ability to induce autophagy. Uridine 5′-diphospho (UDP)-glucuronosyltransferase (UGT)1A induction is one of the mechanisms that is responsible for the cancer chemopreventive activity of SFN. The current study demonstrates that rapamycin may enhance the chemopreventive effects of SFN on Caco-2 cells; this may be partially attributed to nuclear translocation of nuclear factor erythroid 2-related factor 2 (Nrf2)- and human pregnane X receptor (hPXR)-mediated UGT1A1, UGT1A8 and UGT1A10 induction. These results indicate that targeting autophagy modulation may be a promising strategy for increasing the chemopreventive effects of SFN in cases of colon cancer.

## Introduction

Previous epidemiological studies have indicated that dietary intake of cruciferous vegetables may protect against multiple cancers and their protective effects are partially credited to chemopreventive phytochemicals, particularly isothiocyanates (ITCs) (1,2). Sulforaphane (4-methylsulfinylbutyl ICT; SFN), a significant type of ITC, is formed by the hydrolysis of its glucosinolate precursor, glucoraphanin, which is highly enriched in cruciferous vegetables. As a chemopreventive compound that interferes with various cancers, SFN targets the different stages of carcinogenesis, including initiation, promotion and progression (3). During cancer initiation, SFN may enhance the detoxification and removal of carcinogens by inducing phase II enzymes, and blocking carcinogen activation via the inhibition of phase I enzymes. Furthermore, SFN may block the promotion and progression of carcinogenesis by regulating various signaling pathways to suppress cell growth and induce cell death (4).

With respect to phase I enzyme metabolism, SFN has been demonstrated to decrease the enzyme activities of cytochromes P450 (CYPs) 1A1 and 2B1/2 in rat hepatocytes, as well as CYP3A4 in human hepatocytes (5). Zhou *et al* (6) further revealed that SFN is an antagonist of human pregnane X receptor (hPXR) and inhibits hPXR-mediated CYP3A4 expression in human primary hepatocytes. The majority of SFN studies have focused on phase II enzyme induction via the Keap1-nuclear factor erythroid 2-related factor 2 (Nrf2)-antioxidant response element (ARE) signaling pathways. SFN has been demonstrated to induce phase II enzymes, namely glutathione-S-transferase (GST), nicotinamide adenine dinucleotide phosphate quinine oxidoreductase and uridine 5′-diphospho (UDP)-glucuronosyltransferase (UGT) (3). UGT, the representative member of the phase II enzyme family, conjugates endogenous and exogenous substrates with a β-glucuronic acid moiety. Following glucuronidation, these compounds are more water soluble and easily excreted (7). UGT1A1 is a major isoform of the UGT1A family and is the only isoform with bilirubin glucuronidating activity in humans. In addition to bilirubin glucuronidation, numerous therapeutic agents and mutagenic xenobiotics are also substrates of UGT1A1 (8). UGT1A8 and UGT1A10 have been demonstrated to be specifically expressed in the human intestine, and are significant in carcinogen metabolism, as is UGT1A1, which is expressed in the epithelium of colorectal tissues (9).

Autophagy is a protein degradation pathway by which macromolecules and organelles are transported to lysosomes for degradation. This process has been implicated in physiological conditions, such as protein turnover, and in certain diseases, such as neurodegenerative disorders, infectious diseases and cancer (10,11). Due to the cancer-associated changes in autophagy induction, numerous studies focus on the pharmacological manipulation of autophagy for cancer prevention or therapy. Various autophagy activators and inhibitors of autophagy are currently under investigation to combat cancer, and autophagy inhibition in particular is considered to be a promising strategy for cancer treatment (12). Previous studies have also revealed that SFN may induce autophagy in human prostate (13), colon (14), and breast (15) cancer cells and that SFN-induced apoptosis was prevented by its induction of autophagy. Consequently, in these studies, the cells were co-treated with the autophagy inhibitor, 3-methyladenine (3-MA) or bafilomycin A1 to potentiate SFN-induced apoptosis.

The above-mentioned studies of SFN predominantly focus on cell growth arrest or cell death, which were induced by co-treatment with SFN and autophagy modulators; the studies aimed to promote the chemotherapeutic effects in cancer cases. However, the aim of the current study is to elucidate the chemopreventive properties of SFN, which may be exploited to decrease the incidence of cancer. As described, SFN may reduce the exposure of cells to carcinogens via the induction of phase II enzymes and inhibition of phase I enzymes at the cancer initiation stage. Therefore, the present study investigated whether the addition of autophagy modulators exerts changes on phase II enzymes, and evaluated the mechanism of changes in expression. In the present study, the expression of UGT1A1, UGT1A8, and UGT1A10 (three representative members of the phase II enzyme family) and transcription factors, Nrf2 and hPXR, was analyzed in cells co-treated with SFN and rapamycin (an activator of autophagy). In addition, the expression of the phase I enzyme, CYP3A4 (an hPXR target gene) was assayed in cells co-treated with SFN and rapamycin. Experiments were performed in the human colon cancer cell line, Caco-2, an effective model for investigating UGT expression and the hPXR signaling pathway (16–18).

## Materials and methods

### Reagents

SFN (D,L-Sulforaphane) and 3-MA were purchased from Sigma-Aldrich (St. Louis, MO, USA). Rapamycin was purchased from Cell Signaling Technology (Beverly, MA, USA). Dulbecco’s modified Eagle’s medium (DMEM), and fetal bovine serum (FBS) were acquired from Gibco (Rockville, MD, USA) and cell counting kit-8 (CCK-8) was acquired from Dojindo Laboratories (Kumamoto, Japan). The primary rabbit anti-human polyclonal antibody against microtubule-associated protein 1 light chain 3 (LC3) was purchased from Cell Signaling Technology and the rabbit anti-human polyclonal anti-Nrf2 primary antibody was manufactured by Santa Cruz Biotechnology (Santa Cruz, CA, USA). The mouse anti-human monoclonal anti-β-actin primary antibody and fluorescein isothiocyanate (FITC)-conjugated monoclonal goat anti-rabbit secondary antibodies were purchased from ZSGB-Biotechnology, Co., Ltd. (Beijing, China).

### Cell culture

The human colon Caco-2 adenocarcinoma cells (Shanghai Institutes for Biological Science, Chinese Academy of Sciences; Shanghai, China) were maintained as monolayers in DMEM supplemented with 20% non-heat-inactivated FBS, 100 U/ml penicillin and 100 μg/ml streptomycin at 37°C in a humidified atmosphere of 5% CO_2_. The cells used in the experiments were in the logarithmic growth phase. A stock solution of SFN was initially prepared in dimethyl sulfoxide (DMSO) and further diluted to the required concentration in the culture medium. An equal volume of DMSO was also added to the control cells (DMSO-treated control; 0 μM SFN). The final DMSO concentration in the culture medium was maintained at <0.1%.

### CCK-8 cytotoxicity assay

Caco-2 cells were seeded into flat-bottomed 96-well culture plates at a density of 8×10^3^ per well and allowed to adhere for 24 h at 37°C. The cells were then treated with 2.5, 5 and 10 mM of 3-MA, or 10 nM rapamycin, in the presence or absence of 25 μM SFN for 24 h. One group of cells was treated with only 25 μM SFN. Two control groups were included in the experiments: One without drug treatment and one without cells. Cell cytotoxicity was assessed using the CCK-8 kit according to the manufacturer’s instructions. Cells were incubated in the culture medium containing the CCK-8 reagent at 37°C for 2 h, and the absorbance of the solution at a wavelength of 450 nm was determined using a microplate spectrophotometer (Lambda 850; PerkinElmer, Waltham, MA, USA). All experiments were performed in quadruplicate wells. The rate of inhibition of cell viability was calculated according to the following formula: Inhibition rate of cell viability (%) = [1–A450 (sample)/A450 (control)] × 100.

### Western blot analysis of LC3 expression

Caco-2 cells were treated with SFN at a range of concentrations (0, 5, 15, 25 and 35 μM) for 24 h, and treated with SFN at 25 μM for varying lengths of time (6, 12, 24 and 36 h). In addition, the cells were treated with autophagy modulators alone (2.5 mM 3-MA or 10 nM rapamycin) or in combination with 25 μM SFN for 24 h. Cells were then washed twice in ice-cold phosphate-buffered saline (PBS) and lysed in complete cell lysis buffer (50 mM Tris-HCl [pH 7.4], 150 mM NaCl, 1% Triton X-100, 0.25% sodium deoxycholate, 1 mM EDTA, 1 mM NaF, 1 mM dithiothreitol, 1 mM phenylmethylsulfonyl fluoride, 1 mM activated Na3VO4, 1 μg/ml aprotinin, 1 μg/ml leupeptin and 1 μg/ml pepstatin; Beyotime Institute of Biotechnology, Beijing, China). The protein concentration of the cell lysate was determined using the bicinchoninic acid assay (Hyclone-Pierce, Logan, UT, USA). Proteins were resolved by SDS-PAGE and transferred to polyvinylidene difluoride (PVDF) membranes. The membranes were blocked in 5% nonfat dry milk containing 0.1% Tween-20 at room temperature for 1 h, and then probed with the primary antibodies against LC3 (1:1,000) and β-actin (1:2,000) overnight at 4°C. Following washing, the PVDF membranes were incubated with horseradish peroxidase-conjugated goat anti-rabbit polyclonal secondary antibody (1:5,000; Dako, Glostrup, Denmark). The bands were detected using enhanced chemiluminescence and the intensities of acquired bands were evaluated using a computerized image analysis system (Kodak MI software; Carestream Health UK Ltd., Hertfordishire, UK) and normalized to β-actin (the endogenous control).

### Localization of LC3 and Nrf2 by immunocytochemistry

Caco-2 cells were plated on sterile coverslips in a 6-well plate at a density of 1.5×10^5^ cells per well. The cells were divided into the six groups as described in the cytotoxic assay subsection. Following fixing with 4% paraformaldehyde, the cells were permeabilized with 0.3% Triton X-100 for 5 min and blocked with 5% normal goat serum for 1 h at 25°C. Cells were subsequently incubated with the rabbit anti-LC3 (1:200) or rabbit anti-Nrf2 (1:100) primary antibodies overnight at 4°C. Following washing with PBS, the cells were immunostained with FITC-conjugated goat anti-rabbit secondary antibody (1:100) for 1 h at room temperature. The nuclei of the cells were stained with 4′,6-diamidino-2-phenylindole. Immunofluorescence images were acquired using a Carl Zeiss LSM710 confocal laser microscope (Carl Zeiss, Oberkochen, Germany; objective lens magnification, ×63).

### Ultrastructures observed by transmission electron microscopy

On entering the logarithmic phase, the Caco-2 cells were divided into six treatment groups: 0 μM SFN (DMSO-treated control); 25 μM SFN; 2.5 mM 3-MA; 10 nM rapamycin; 25 μM SFN plus 2.5 mM 3-MA; and 25 μM SFN plus 10 nM rapamycin. Following 24 h of treatment, the cells were washed with PBS, collected by centrifugation using a Minispin centrifuge (Eppendorf, Hamburg, Germany) at 1,000 × g and fixed in ice-cold 2.5% electron microscopy grade glutaraldehyde. The specimens were subsequently rinsed with 0.1 M PBS, postfixed in 1% osmium tetroxide (Absin Bioscience Inc., Beijing, China), dehydrated through a graded series of ethanol solutions (30–90%) and processed for Epon™ embedding (Momentive Specialty Chemicals, Inc., Columbus, OH, USA). Semi-thin sections (600–800 nm) were stained with toluidine blue and representative areas were selected for ultra-thin sectioning. Ultra-thin sections (50–70 nm) stained with uranyl acetate and lead citrate were examined with a JEM-1011 electron microscope (Jeol Ltd., Tokyo, Japan).

### Quantitative reverse transcription polymerase chain reaction (RT-qPCR)

Caco-2 cells were divided into the six above-mentioned groups and total RNA was isolated from the cells using TRIzol reagent (Invitrogen Life Technologies, Carlsbad, CA, USA) according to the manufacturer’s instructions. Reverse transcription was conducted with 1 μg RNA, oligo-dT15 primer and Moloney murine leukemia virus reverse transcriptase (Toyobo, Osaka, Japan) in a volume of 20 μl. The levels of UGT1A1, UGT1A8, UGT1A10, hPXR and CYP3A4 transcripts were analyzed by RT-qPCR, with SYBR^®^ Green fluorescence (Toyobo) detected using the LightCycler^®^ system (LightCycler 2.0; Roche, Basel, Switzerland). In all samples the levels of the reference gene, β-actin served as an internal control for the normalization of RNA loading and quality differences. The qPCR reaction conditions were as follows: An initial step of 30 sec at 95°C, followed by 45 cycles for 5 sec at 95°C, 10 sec at 58°C and 15 sec at 72°C. The experiments were performed in triplicate. Relative mRNA levels were calculated as the ratio between the target mRNA level and the corresponding internal control (β-actin).

Gene-specific primer sequences were acquired from PrimerBank (http://pga.mgh.harvard.edu/primerbank/index.html) and are shown in Table I. All primers were synthesized by BioSune (Shanghai, China) following BLAST (http://blast.ncbi.nlm.nih.gov/Blast.cgi) searches to ensure their target specificity. Gene-specific amplifications were identified by analyzing the melting curve data and visualizing RT-qPCR products by agarose gel electrophoresis.

### Statistical analysis

Statistical analysis was performed using SPSS version 17.0 (SPSS, Inc., Chicago, IL, USA). Data are from a minimum of three independent experiments and are expressed as the mean ± standard error. P<0.05 was considered to indicate a statistically significant difference.

## Results

### Probing the cytotoxicity of SFN and autophagy modulators in Caco-2 cells

**Figure 1:**
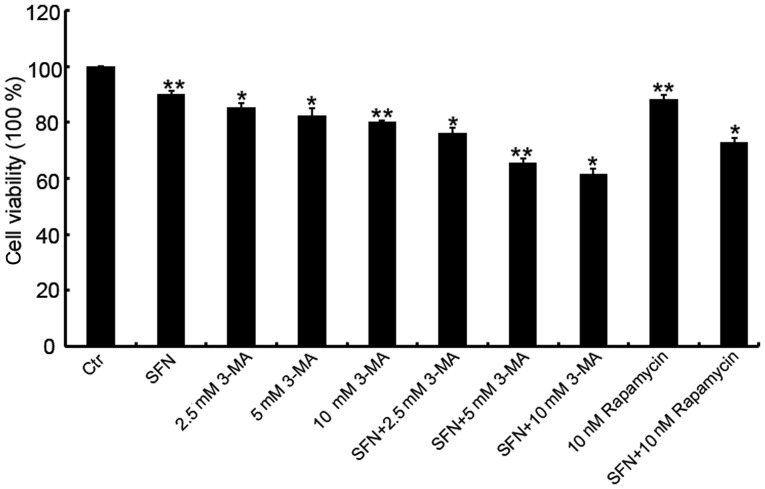
Inhibition rate of cell viability of Caco-2 cells was measured using the cell counting kit-8 assay. Cells were treated with SFN (25 μM), 3-MA (2.5, 5 and 10 mM) and rapamycin (10 nM). The combination of SFN (25 μM) with 3-MA (2.5, 5, and 10 mM) or rapamycin (10 nM) was also measured in Caco-2 cells. The duration of all treatments was 24 h. Ctr, control; SFN, sulforaphane; 3-MA, 3-methyladenine.^*^P<0.05 and ^**^P<0.01.

To limit the changes in viability due to therapeutic agent-induced cytotoxicity, the appropriate therapeutic agent concentration was selected to be used in future experiments by assessing the Caco-2 cell cytotoxicity of SFN and 3-MA at various concentrations with the CCK-8 assay. Compared with the cytotoxicity of 25 μM SFN treatment alone, co-treatment with 3-MA exhibited a higher cytotoxicity, and a dose-dependent increase in the inhibition rate of cell viability was observed when 25 μM SFN was combined with 2.5, 5 or 10 μM 3-MA (Fig. 1). To reduce the influence of cytotoxicity on cell viability, a concentration of 2.5 μM 3-MA was used in combination with 25 μM SFN in subsequent experiments due to its low inhibition rate (23.9%).

The concentration of rapamycin (10 nM) that was used in the experiments was selected based on the manufacturer’s recommendation. The cytotoxicity of the rapamycin/SFN combination treatment was further investigated and the results demonstrated that this combination exhibited a relatively low inhibition rate (26.9%; Fig. 1).

### SFN, 3-MA and rapamycin modulate autophagy

The process of autophagy commences with the formation of double-membrane vacuoles (autophagosomes), which enclose cytoplasmic material. Autophagosomes subsequently fuse with late endosomes and/or lysosomes to mature into autolysosomes, where the inner membrane and contents are degraded. To demonstrate the autophagy-induction effect of SFN on colon cancer Caco-2 cells, and investigate the appropriate concentration and duration of SFN treatment, the autophagy induction was analyzed by examining the conversion of LC3-I to LC3-II, which is the conventional approach for monitoring autophagy induction. LC3 is an autophagosome marker that exists in two forms: LC3-I, a 16-kDa cytosolic protein, and LC3-II, a processed 14-kDa form localized in the outer and inner membranes of the autophagosome (19). SFN and rapamycin are autophagy activators while 3-MA inhibits autophagy at an early stage. Therefore, the levels LC3-II expression positively correlate with their degrees of autophagy induction, however, an enhancement of LC3 expression may also indicate inhibition of the autophagic process at a later stage.

**Figure 2:**
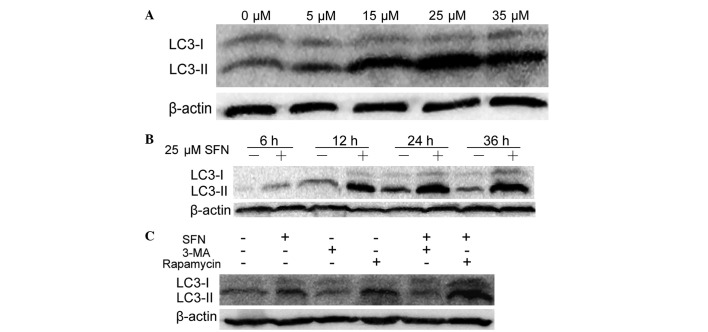
Western blot analysis of LC3-I and -II using lysates from Caco-2 cells following treatment with SFN or SFN combined with autophagy modulators. (A) Caco-2 cells treated with 0, 5, 15, 25 and 35 μM SFN for 24 h. (B) Caco-2 cells were treated with 0 or 25 μM SFN for 6, 12, 24 and 36 h. (C) Caco-2 cells were treated with 0 μM SFN (dimethyl sulfoxide-treated control), 25 μM SFN, 2.5 mM 3-MA or 10 nM rapamycin in the presence or absence of 25 μM SFN for 24 h. The gel shown is representative of three independent experiments. LC3, protein 1 light chain 3; SFN, sulforaphane; 3-MA, 3-methyladenine.

As shown in Fig. 2A, autophagy was induced by SFN, as a dose-dependent increase in LC3-II levels was observed in Caco-2 cells that were treated with SFN ranging from 0 μM (DMSO-treated control) to 25 μM; however, the levels of LC3-II protein diminished when the concentration was increased to 35 μM. The lysates of Caco-2 cells were subsequently subjected to western blot analysis following treatment with 25 μM SFN or DMSO for 6, 12, 24 and 36 h; the LC3-II levels increased in a time-dependent manner, in the presence of 25 μM SFN, from 0–36 h (Fig. 2B). These results indicated that SFN induced autophagy in a dose- and time-dependent manner in Caco-2 cells.

3-MA and rapamycin are known autophagy modulators; 3-MA has been demonstrated to inhibit autophagy at an early stage, causing a reduction in autophagosome formation. Rapamycin is an autophagy activator that inhibits the mammalian target of rapamycin complex (11). To investigate the effects of 3-MA and rapamycin on autophagy in Caco-2 cells, their impact on LC3 conversion was analyzed. As shown in Fig. 2C, compared with the DMSO-treated control, 3-MA reduced LC3-II protein expression levels while rapamycin enhanced its expression levels. Compared with cells treated only with SFN, the 3-MA/SFN combination treatment reduced LC3-II expression levels and the rapamycin/SFN combination treatment enhanced the expression levels. These results are consistent with the above-mentioned inhibitory and activation effects of 3-MA and rapamycin, respectively on autophagy regulation.

**Figure 3:**
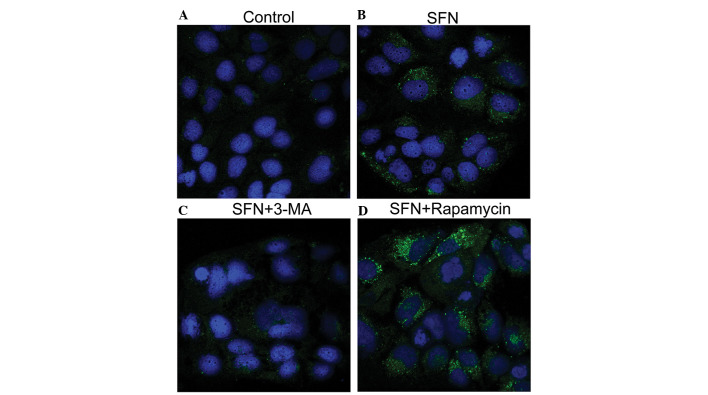
Immunocytochemical analysis of protein 1 light chain 3 localization in Caco-2 cells treated for 24 h with (A) 0 μM SFN (dimethyl sulfoxide control), (B) 25 μM SFN, (C) 2.5 mM 3-MA or (D) 10 nM rapamycin combined with 25 μM SFN. Images are representative of three independent experiments (magnification, ×630). SFN, sulforaphane; 3-MA, 3-methyladenine.

The intracellular localization of LC3 was visualized by immunofluorescence microscopy to further establish that autophagy is modulated by the combination treatment of Caco-2 cells with SFN and 3-MA or rapamycin. As shown in Fig. 3, the DMSO-treated control Caco-2 cells exhibited weak and diffusely distributed LC3-associated green fluorescence, whereas SFN treatment altered the distribution, with coarse dots and punctate staining observed. This LC3-positive punctate staining (LC3-II) is a typical feature of LC3 distribution within autophagosomes (11). Furthermore, this punctate staining was diminished by the addition of 3-MA, while an increased quantity of LC3-positive punctates were observed as a result of SFN/rapamycin combination treatment. These results are largely consistent with the levels of LC3-II protein that were observed by western blot analysis (Fig. 2C).

**Figure 4:**
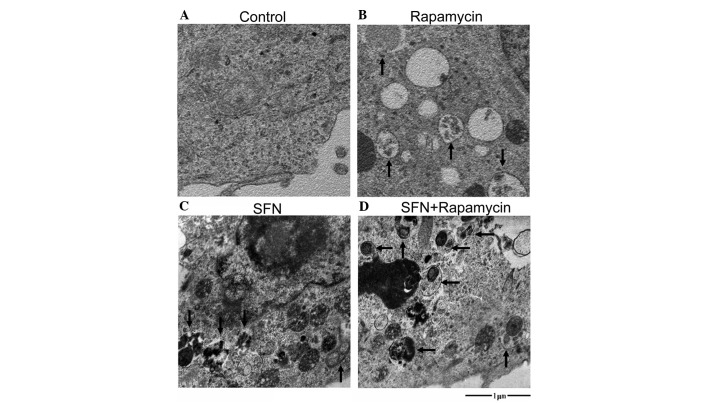
Transmission electron microscopy of ultrastructures of Caco-2 cells treated for 24 h with (A) 0 μM SFN (dimethyl sulfoxide control), (B) 10 nM rapamycin, (C) 25 μM SFN and (D) 25 μM SFN and 10 nM rapamycin. The arrows indicate autophagosomal or autolysosomal vacuoles (magnification, ×15,000). SFN, sulforaphane.

In addition, the ultrastructures of the cells were examined by transmission electron microscopy (Fig. 4). Numerous membranous vacuoles, autophagosomes and autolysosomes (indicated by the arrows), containing residual digested material, appeared in the cytoplasm of the SFN-, rapamycin- and SFN/rapamycin-treated cells, while relatively few corresponding structures were observed in the cytoplasm of the DMSO-treated control cells or in the groups of cells that were treated with 3-MA alone or SFN/3-MA in combination (data not shown). The observation of autophagic vacuoles further demonstrates the effects of SFN and rapamycin on autophagy.

### Rapamycin modulates the induction of UGT1A isoform expression by SFN

**Figure 5:**
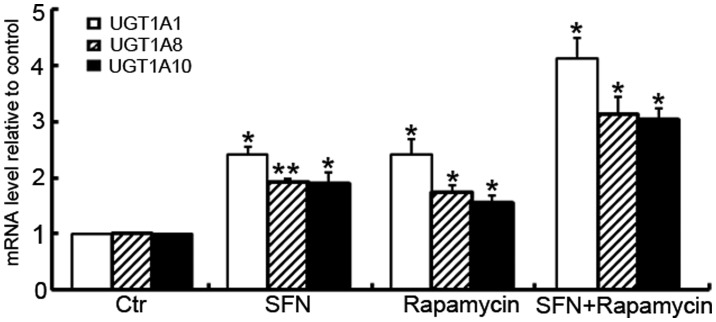
Quantitative reverse transcription polymerase chain reaction analysis of UGT1A1, UGT1A8 and UGT1A10 at the mRNA level in Caco-2 cells. Cells were treated for 24 h with 0 μM SFN (dimethyl sulfoxide control), 25 μM SFN, 10 nM rapamycin or 10 nM rapamycin plus 25 μM SFN. Results are presented as the mean ± standard error. n=3; ^*^P<0.05 and ^**^P<0.01. Ctr, control; SFN, sulforaphane.

To further investigate the modulation of rapamycin on UGT1A isoform expression by SFN, the mRNA levels of the UGT1A isoforms for the SFN-, rapamycin- and SFN/rapamycin-treated cells were analyzed using RT-qPCR. As shown in Fig. 5, similar trends were identified in the induction of UGT1A isoforms, when Caco-2 cells were treated with these small molecules. SFN-induced mRNA expression of UGT1A1, UGT1A8 and UGT1A10 was further enhanced in the presence of rapamycin (P=0.001, P=0.002 and P=0.003, respectively).

### Nrf2 nuclear translocation in Caco-2 cells

**Figure 6:**
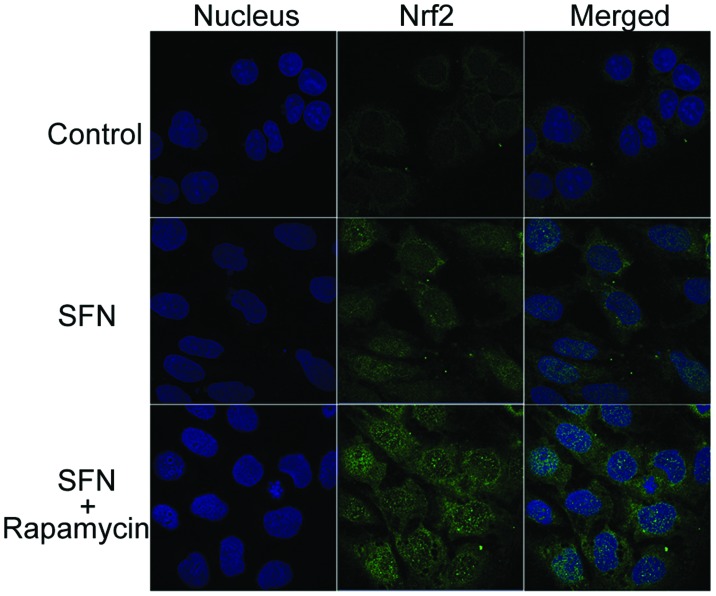
Induction of Nrf2 nuclear translocation in Caco-2 cells was examined by immunocytochemistry. Cells were treated for 24 h with 25 μM SFN, 10 nM rapamycin, or a combination of the two. Images are representative of three independent experiments (magnification, ×630). Nrf2, nuclear factor erythroid 2-related factor 2; SFN, sulforaphane.

Under basal conditions, Nrf2 is sequestered in the cytoplasm by Keap1 and, therefore, is not involved in the induction of phase II enzymes. The interaction between Nrf2 and Keap1 is perturbed in response to chemical or oxidative stress, resulting in the translocation of Nrf2 into the nucleus (20). Nrf2 may then bind to the AREs to stimulate the transcription of phase II enzymes, including UGT1A isoforms (21). Therefore, in the present study, Nrf2 localization to the cytoplasm and nucleus of Caco-2 cells was monitored by immunocytochemistry. As presented in Fig. 6, a small quantity of Nrf2 protein (indicated by green fluorescence) was restricted to the cytoplasm in the DMSO-treated control. Treatment with SFN alone caused the accumulation of Nrf2 in the nuclear region, with SFN/rapamycin combination treatment resulting in elevated Nrf2 protein staining, particularly increased Nrf2 nuclear staining. This indicated that the transcription factor, Nrf2 may be significant in the induction of UGT1A1, UGT1A8 and UGT1A10 expression. Caco-2 cells that were treated with rapamycin alone (data not shown) exhibited similar results to the DMSO-treated control, as Nrf2 only exhibited a marginal quantity of cytosolic fluorescence.

### Modulation of hPXR mRNA expression by rapamycin

**Figure 7:**
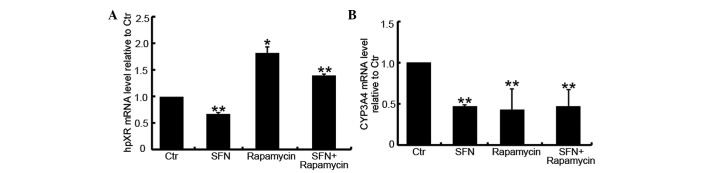
Induction of (A) hPXR and (B) CYP3A4 mRNA expression in Caco-2 cells as analyzed by quantitative reverse transcription polymerase chain reaction. Cells were treated for 24 h with 25 μM SFN, 10 nM rapamycin or a combination of the two. Results are presented as the mean ± standard error. n=3; ^*^P<0.05 and ^**^P<0.01. Ctr, control; SFN, sulforaphane.

As demonstrated by the findings of the present study, the addition of rapamycin enhances the SFN-induced expression of UGT1A isoforms. Rapamycin treatment alone was also observed to induce UGT1A1 expression when compared with DMSO-treated control cells. However, no apparent Nrf2 nuclear translocation was observed in Caco-2 cells that were treated with rapamycin. In addition to the activation of the Keap1-Nrf2-ARE signaling pathway, the expression of UGT1A isoforms may also be induced by the activation of hPXR (18,22). Therefore, the levels of hPXR mRNA were investigated in Caco-2 cells treated with SFN, rapamycin or a combination of the two. As shown in Fig. 7A, compared with the DMSO-treated group, SFN inhibited the expression of hPXR mRNA (P=0.024) while rapamycin enhanced its expression (P<0.001). Furthermore, the SFN/rapamycin combination treatment group exhibited an increased level of hPXR mRNA (P=0.008).

### Modulation of CYP3A4 mRNA expression by rapamycin

hPXR is a key transcription factor responsible for the induction of CYP3A4 expression (23), and Zhou *et al* (6) reported that SFN may inhibit hPXR-mediated CYP3A4 expression and CYP3A4-dependent drug clearance. As rapamycin increases the mRNA expression of hPXR, the present study evaluated whether this small molecule influences CYP3A4 expression. As shown in Fig. 7B, compared with the DMSO-treated control group, SFN-, rapamycin- and SFN/rapamycin-treated cells exhibited reduced levels of CYP3A4 mRNA (P<0.001), however, no significant differences were identified between these three groups (P>0.05). This indicated that the induction of hPXR by rapamycin does not lead to increased levels of CYP3A4 expression in Caco-2 cells.

## Discussion

SFN has been demonstrated to activate autophagy in human prostate (13), colon (14), breast (15) and pancreatic carcinoma cells (24). The present study demonstrated that a dose-dependent increase in LC3-II expression levels may be induced in Caco-2 cells by treatment with SFN at concentrations ranging from 5 to 25 μM, and LC3-II levels may be increased in a time-dependent manner by incubation with 25 μM SFN for 6–36 h. In addition, the results indicate that SFN induces autophagy in a dose- and time-dependent manner in Caco-2 cells. Based on these results, 25 μM and 24 h were selected as the concentration and duration of SFN treatment in the subsequent experiments. To evaluate the potential role of autophagy in SFN-mediated cancer chemoprevention, Caco-2 cells were treated with SFN in combination with 3-MA or rapamycin. Initially, the cytotoxicity of the co-treatment on Caco-2 cells was assessed using a CCK-8 kit. In previous reports, 5 or 10 mM of 3-MA were used to potentiate apoptosis, which is induced by chemoprevention or chemotherapy agents (13,14,25,26). Therefore, the cytotoxicity of 25 μM SFN combined with 2.5, 5 or 10 μM of 3-MA, as well as with 10 nM rapamycin (as recommended by the manufacturer) was investigated. Based on the low level of cytotoxicity observed in Caco-2 cells, concentrations of 2.5 μM 3-MA and 10 nM rapamycin were selected in combination with 25 μM SFN for subsequent experiments. The ability of 3-MA and rapamycin to inhibit or activate autophagy, respectively, in Caco-2 cells was demonstrated by the conversion of LC3-I to LC3-II (as shown by western blot and immunocytochemical analysis), and was also established by the observed formation of autophagosomes and autolysosomes by transmission electron microscopy.

In prostate, colon and breast cancer cells, the addition of 3-MA or bafilomycin A1 potentiates SFN-induced apoptotic cell death (15). In the present study, co-treatment with 3-MA or rapamycin was observed to aggravate the cytotoxicity of SFN on Caco-2 cells. In pancreatic carcinoma (PC) cells (24), the blockage of autophagy by chloroquine or the induction of autophagy by rapamycin did not affect the cell viability of SFN-treated PC cells, indicating that changes in autophagy do not influence SFN-induced cell death in PC cells. This contradiction may be due to the differences between cancer cell lines and autophagy inhibitors that were analyzed.

The majority of previous studies regarding the cancer chemopreventive properties of SFN focused on its chemopreventive effects at the promotion and progression stages in carcinogenesis, as well as attempting to determine whether the regulation of autophagy (such as by co-treatment with autophagy modulators) may promote SFN-induced apoptosis in cancer cells. The current study focussed on elucidating the SFN-mediated chemopreventive effects at the cancer initiation stage, and investigated the role of autophagy in phase I and phase II enzyme expression. In a previous study, SFN at hypotoxic levels (10–30 μM) was observed to induce UGT1A in a dose-dependent manner, while 25 μM SFN treatment for 24 h enhanced UGT1A1, UGT1A8 and UGT1A10 mRNA expression in Caco-2 cells (27). To the best of our knowledge, the present study is the first to report that rapamycin increases the mRNA expression level of UGT1A1, and that co-treatment with rapamycin and SFN results in a synergistic induction of UGT1A1, UGT1A8 and UGT1A10 expression at the mRNA level. Conversely, 3-MA exhibited no influence on UGT1A isoform expression levels, and the addition of 3-MA attenuated the SFN-induced mRNA expression levels of the UGT1A isoforms.

The potential mechanisms by which UGT1A isoform expression is regulated by autophagy modulators alone or in combination with SFN was subsequently investigated. Studies have demonstrated that several ligand-activated transcription factors regulate the induction of UGTs, including Nrf2, aryl hydrocarbon receptor, constitutive androstane receptor (CAR), peroxisome proliferator-activated receptor-α and PXR (7,28). In our previous study, SFN was observed to induce Nrf2 nuclear translocation and activation in Caco-2 cells. The present study demonstrated that co-treatment with SFN and rapamycin exhibited higher Nrf2 intracellular expression levels and a more intense nuclear staining than was observed with SFN treatment alone. This indicates that the addition of rapamycin may enhance UGT1A isoform expression levels through an increase in Nrf2 activation.

However, while rapamycin treatment alone enhanced UGT1A1 expression levels in Caco-2 cells, it did not upregulate Nrf2 intracellular expression levels or activate Nrf2 nuclear translocation. This indicated that other transcription factors may be participating in the rapamycin-mediated induction of UGT1A isoforms. Certain reports have proposed that UGT1A1, UGT1A3, UGT1A4 and UGT1A6 contain functional PXR response elements and are inducible via PXR-specific ligands (18,22,29). Gardner-Stephen *et al* (18) reported that UGT1A1 was markedly upregulated by the transfection of hPXR variants in Caco-2 cells, and the results of UGT1A8 and UGT1A10 were not consistent among replicates. Therefore, hPXR mRNA transcription was examined in the present study using RT-qPCR. Rapamycin was shown to upregulate hPXR mRNA levels, while SFN suppressed its expression, which was consistent with a previous report (6). These results indicated that the induction of UGT1A1 gene expression by rapamycin is partially attributed to signaling through the hPXR pathway, while SFN induces UGT1A1 gene expression, including the expression of UGT1A8 and UGT1A10, predominantly through the Nrf2 signaling pathway and independently of hPXR regulation. In the present study, the mRNA expression of UGT1A8 and UGT1A10 was found to be enhanced by rapamycin treatment alone, however, this effect was not significant when compared with the control group, which may have been due to the upregulation of hPXR. Therefore, the combination of SFN and rapamycin appears to synergistically induce UGT1A1 gene expression in Caco-2 human colon cancer cells via the simultaneous stimulation of the Nrf2 and hPXR signaling pathways. However, the potential underlying mechanism of hPXR involvement in the enhancement of UGT1A8 and UGT1A10 expression requires further investigation.

CYP3A4 is the major CYP isoform that is present in the human liver and small intestine. It is regulated by PXR and CAR at the transcriptional level (30) and contributes to the biotransformation of >50% of current prescription medications (31). CYP3A4 induction is a common mechanism of adverse drug-drug interactions. In addition, certain procarcinogens require CYP3A4-mediated metabolic activation and are converted into highly reactive intermediates that promote carcinogenesis. For example, CYP3A4 expression is the most important determinant of the hepatic carcinogen, aflatoxin B1 (AFB1) conversion into the AFB, 8,9-epoxide, which binds to DNA to form an AFB1-DNA adduct (32,33). Thus, SFN as an antagonist of hPXR may reduce adverse therapeutic agent responses and enhance cancer chemoprevention via the repression of hPXR-regulated CYP3A4. In the current study, the addition of rapamycin did not influence SFN-regulated CYP3A4 expression, however, it was observed to enhance the expression of hPXR at the mRNA level. Therefore, with regards to CYP3A4 expression, co-treatment with rapamycin may not affect the chemopreventive property of SFN, which involves the inhibition of CYP3A4. The results also indicate that in addition to hPXR, other signaling pathways, which were inhibited by co-treatment with rapamycin and SFN are involved in CYP3A4 expression. However, CYP3A4 is also particularly important for the detoxification of xenobiotic chemicals, and the excessive suppression of CYP3A4 expression may lead to negative impacts on normal metabolism.

In conclusion, co-treatment with rapamycin may enhance SFN-induced autophagy and UGT1A1, UGT1A8 and UGT1A10 expression levels. The underlying mechanism of the synergistic induction of UGT1A isoforms may be associated with the combined activation of Nrf2 and hPXR signaling pathways. Furthermore, the upregulation of hPXR did not lead to CYP3A4 induction, which is associated with adverse therapeutics agent responses and procarcinogen activation. However, a limitation of the current UGT1A isoform and CYP3A4 study is that these enzymes were only analyzed with regard to their mRNA levels. Further information may be obtained by analyzing the metabolic activity of enzymes in Caco-2 cells following co-treatment with SFN and rapamycin. The current study provides evidence supporting the potential use of an autophagy activator for the enhancement of the chemopreventive effects of SFN, particularly in the induction of phase II enzymes. However, further studies are required to demonstrate the efficacy of this strategy in healthy cells and *in vivo*.

## Figures and Tables

**Table I:** Primer sequences for quantitative reverse transcription polymerase chain reaction.

Gene name	Primer	Primer sequence, 5′→3′
UGT1A1	Forward	TCCCACTTACTGCACAACAAG
	Reverse	GGTCCGTCAGCATGACATCA
UGT1A8	Forward	TTGATGCCTGTGCGTTAATTGT
	Reverse	GGCAACCTATTCCCCTGGC
UGT1A10	Forward	GCCCCGTTCCTTTATGTGTGT
	Reverse	ATCTTCCAGAGTGTACGAGGTT
β-actin	Forward	CATGTACGTTGCTATCCAGGC
	Reverse	CTCCTTAATGTCACGCACGAT
hPXR	Forward	TTGCCCATCGAGGACCAGAT
	Reverse	GTCTCCGCGTTGAACACTGT
CYP3A4	Forward	AAGTCGCCTCGAAGATACACA
	Reverse	AAGGAGAGAACACTGCTCGTG
